# Battling COVID-19 Pandemic: Sphingosine-1-Phosphate Analogs as an Adjunctive Therapy?

**DOI:** 10.3389/fimmu.2020.01102

**Published:** 2020-05-15

**Authors:** Farha Naz, Mohd Arish

**Affiliations:** ^1^Center for Interdisciplinary Research in Basic Science, Jamia Millia Islamia, New Delhi, India; ^2^Jamia Hamdard Institute of Molecular Medicine, Jamia Hamdard, New Delhi, India

**Keywords:** COVID-19, SARS-CoV-2, S1P analogs, cytokine storm, immuno-modulators

## Abstract

With the sudden outbreak of COVID-19 patient worldwide and associated mortality, it is critical to come up with an effective treatment against SARS-CoV-2. Studies suggest that mortality due to COVID 19 is mainly attributed to the hyper inflammatory response leading to cytokine storm and ARDS in infected patients. Sphingosine-1-phosphate receptor 1 (S1PR1) analogs, AAL-R and RP-002, have earlier provided *in-vivo* protection from the pathophysiological response during H1N1 influenza infection and improved mortality. Recently, it was shown that the treatment with sphingosine-1-phosphate receptor 1 analog, CYM5442, resulted in the significant dampening of the immune response upon H1N1 challenge in mice and improved survival of H1N1 infected mice in combination with an antiviral drug, oseltamivir. Hence, here we suggest to investigate the possible utility of using S1P analogs to treat COVID-19.

## Introduction

Severe acute respiratory syndrome coronavirus 2 (SARS-CoV-2), emerged from Wuhan, China, has now become a threat to the whole world. The disease associated with SARS-CoV-2 is termed as coronavirus disease-2019 (COVID-19). World Health Organization (WHO) has now declared COVID-19 as a global pandemic affecting more than 200 countries, resulting in 1991,562 confirmed cases and 130, 885 deaths, as of 16 April 2020, and still counting (1). The main reason for disease severity in COVID-19 is due to aberrant and excessive cytokine production, leading to pathophysiology called cytokine storm, and Acute Respiratory Distress Syndrome (ARDS) (2–4). The serum of infected patients showed increased levels of pro-inflammatory cytokines such as IL-2, TNF-α, IL-1β, IFN-γ, MCP-1, and MIP1A, resulting in cytokine storm (4). Building on the above observation, these patients were given corticosteroids to reduced inflammation-induced lung damage. However, previous studies with the influenza virus suggest that adjunctive corticosteroid therapy rather increase mortality. Also, corticosteroid therapy in patients with Middle East respiratory syndrome (MERS) resulted in delayed viral clearance (5), altogether suggesting the cautious use of corticosteroids in COVID-19 patients. Hence, due to the limited effectiveness of corticosteroids, the use of alternate immuno-modulators could be suggested at present.

## Coronavirus (CoV) Induced Immuno-Pathology

Before the emergence of novel SARS-CoV-2 in late 2019, MERS-CoV, and SARS-CoV were considered to be highly pathogenic. These two CoV contribute to acute lung pathology as a result of cytokine storm in infected patients, which could be lethal if left untreated (6). The earlier study with SARS-CoV infection in non-human primates, cynomolgus macaques, resulted in increased expression of IFN-α, IFN-β, and IFN- γ, IFN-λ at mRNA level in lungs of the infected macaque (7). Similarly, SARS-CoV infection in BALB/c mice showed increase pro-inflammatory cytokine secretion, eventually leading to lethal acute lung injury and high fatality rate in mice (8). Mouse model of MERS Coronavirus (CoV) infection demonstrated enhanced expression of CCL2, IL-6, and TNF-α at mRNA level in the lungs of infected mice. Comparative *in-vitro* infection studies with SARS-CoV and MERS-CoV showed that both these viruses upregulated the expression of TNF-α, IL-6, and IL-12 at mRNA level in monocytes derived macrophages (9).

In context to SARS-CoV-2, reports are emerging from China, the epicenter of COVID-19, which showed a similar trend in cytokine profile as with SARS-CoV and MERS-CoV (10). In severe cases of COVID-19, patients showed increased serum cytokine levels of IL-2, TNF- α, IL-1β, IFN-γ, MCP-1, MIP1A, and IL-6 (4, 11). Another life-threatening complication, namely ARDS could be developed more often in elderly COVID-19 patients as a result of cytokine storm (4, 12, 13). According to recent research, patients with ARDS have reduced serum S1P levels as compared to healthy controls, which was further associated with non-pulmonary organ failure (14). In this context, detection of serum S1P level in COVID-19 patients may be worth exploring, as it serves as a biomarker for ARDS associated disease severity.

## Therapeutic Potential of S1P Analogs

We have earlier reported therapeutic intervention of using S1P (sphingosine-1-phosphate) analogs during infectious diseases (15, 16). Our unpublished study with S1P analogs in *Mycobacterium tuberculosis* (H37Ra) infected macrophages showed that treatment with S1P analogs results in blunting too much pro-inflammatory response, but also intriguingly leading to clearance of bacterial load. Additionally, S1P signaling was reviewed as a potential target to provide therapeutic benefits in pulmonary disorder (17). As with H1N1 influenza virus infection intra-tracheal AAL-R [(R)-2-amino-4-(4-heptyloxyphenyl)-2-methylbutanol], S1P analog, the treatment showed improved survivability of mice challenged with H1N1 as compared to conventional antiviral therapy. Walsh et al. provide evidence that intra-tracheal administration with AAL-R in infected mice resulted in reduced lung tissue injury as showed by histo-pathological and enzymatic studies (18). The bronchoalveolar lavage (BAL) fluid of these mice revealed reduced pro-inflammatory cytokines such as IFN-α, IL-6, and IFN-γ, and chemokine including CCL2, CCL3, CCL5, CXCL2, and CXCL10. AAL-R treatment doesn't clear the viral load, nevertheless, it doesn't impair host ability to clear viral load, which was supported by the unchanged viral neutralizing antibodies in treated and untreated groups (18). Similarly, RP-002 treatment, functional agonists of S1PR1, reduced mortality of influenza virus-infected mice by reduction in cytokine/chemokines (IFN-α, CCL2, IL-6, and IFN-γ) production (19). The same group later studies the efficacy of RP-002, in a mouse model of the respiratory syncytial virus. Oral administration of RP-002 showed enhanced survival of paramyxovirus PMV infection in mice, as displayed by reduced inflammation in lungs with normal morphology of alveolar sacs of infected mice on RP-002 therapy. Decrease IFN-γ, TNF- α, CCL2, CCL5, CXCL10, IL-1α, and IL-6 secretion was also observed in BAL fluid of RP-002 treated infected mice (20). The authors further revealed that RP-002 treatment reduced CD8^+^ T and Natural killer (NK) cells in the lung infiltrate of infected mice. Lesser number of TNF-α and IL-2 producing IFN-γ^+^ CD8^+^ T cells, after stimulated with immuno-dominant peptides of PMV, was further confirmed in the lymph nodes and lungs of infected mice as compared to mice that received vehicle (20). The previous study with SARS-CoV demonstrates the infiltration of CD8^+^ T cell and NK cells in the lungs of infected mice at the late phase of the infection. Surprisingly, instead of CD8^+^ T cells, CD4^+^ T cells were required for viral clearance, whereas CD8^+^ T cells were rather associated with lung pathology during viral infection (21). Hence, here it is suggested that S1P analogs may block the infiltration of immune cells with inflammatory phenotype, particularly CD8^+^ T cells secreting TNF-α or IFN-γ, which may prevent acute lung injury during COVID-19.

A more recent study by Zhao et al. presented a similar approach of S1PR1 agonist, CYM5542, in providing therapeutic benefits in H1N1 infected mice. Intra-tracheal delivery of CYM5542 results in a marked reduction in lung injury and pro-inflammatory cytokine and chemokines production such as IFN-α, IFN-γ, TNF-α, IL-6, CCL2, CCL3, CCL5, CXCL2, and CXCL10 in BAL fluid of infected mice. Furthermore, the therapeutic efficacy of CYM5542 was improved in the presence of an antiviral drug, oseltamivir (22). As S1P signaling influences myriad of downstream signaling, the role of CYM5542 in the regulation of Mitogen-Activated Protein Kinase (MAPK) and nuclear factor kappa-light-chain-enhancer of activated B cells (NFκB) was studied. CYM5542 treatment results in the reduced phosphorylation status of MAPK such as ERK1/2 and JNK1/2, and p65 subunit of NFκB, resulting in inactivation of the signal and hence cytokine production (22). CYM5542 treatment resulted in the degradation of interferon alpha receptor 1 (IFNAR1), and deactivation signal transducer and activator of transcription 1 (STAT1), thereby limiting IFN- α response (23).

## The Limitations of S1P Analog Therapy

Before recommending S1P as a potential therapy against COVID-19, we must also discuss the possible risk associated with S1P analogs. The common consequence of cytokine storm is acute lung injury that results in ARDS, which proves to be fatal as seen in severe cases of SARS-CoV-2 infected patients. Hence, it is very critical to maintain cytokine homeostasis in response to pulmonary infection by targeting pro-inflammatory immune cells or activating anti-inflammatory pathways (24). However, targeting pro-inflammatory immune cells may not be an advisable approach as it also limits the capacity of the host to clear the infection. Therefore, cautious use of S1P analogs in combination with anti-viral therapy is suggested to ensure clearance of infection without compromising the host defense. It is also worth noting that activation of S1P signaling during *M. tuberculosis* infection has dual action of host protection or disease progression, which depends on stage of *M. tuberculosis* infection. S1P treatment during early infection has profound effect on reduction of infection and disease associated histopathology. On the contrary, S1P treatment during acute *M. tuberculosis* infection exacerbates the disease (25). Therefore, to explore proper therapeutic potential of S1P analogs all these criteria must be taken into consideration. More importantly, S1P analogs have shown therapeutic efficacy in animal model of pulmonary infections, its role in humans has not been studied yet. Most of the human trials related to S1P analogs as immunotherapy have been dedicated to diseases such as Multiple Sclerosis, subacute lupus erythematosus, Crohn's disease, etc. (26). As of now only Fingolimod (FTY720), S1P analog, has been recently approved for clinical trials to test its efficacy against COVID-19. However, due to the broad specificity of FTY720 on S1PR1 and S1PR3-5, more specific S1P analogs such as RP-002 or CYM5542 must also be investigated to minimize off-target effects.

## Concluding Remarks

SARS-CoV-2 induced cytokine storm is a serious immuno-pathology that could lead to the death of infected patients. S1P analogs have earlier protected from pulmonary infection by dampening the cytokine storm (Table 1). Taken together, these reports emphasize the need to consider S1P analogs as potential immuno-modulators in ameliorating SARS-CoV-2 induced cytokine storm. Several studies correlated cytokine storm with lung pathophysiology and have advocated the use of immuno-modulators for therapeutic intervention (27). In this regard, a FDA approved drug for multiple sclerosis, FTY720, which is a S1PR modulator, is recently in the clinical trial to assess its role as an immuno-modulator in COVID-19 (28). As a new vaccine and anti-viral for the treatment of COVID-19 may take more time, alternatively, host-directive therapy could be the current weapon of choice against SARS-CoV-2 (29). Of note, dampening of SARS-CoV-2 induced cytokine storm with S1P analogs warrants further attention in the form of more robust and randomized clinical trials to prove our hypothesis.

**Table 1:** Therapeutic potential of S1P analogs in the suppression of viral induce immuno-pathology.

**S.No**	**S1P analog**	**Viral disease**	**Biological outcome**	**References**
1.	AAL-R	H1N1	Reduced lung tissue injury and pro-inflammatory cytokine secretion	(18)
2.	RP-002	H1N1	Reduced mortality of infected mice as result of reduced proinflammatory cytokines/chemokines production	(19)
3.	RP-002	Paramyxovirus	Reduced inflammation in lungs	(20)
4.	CYM5542	H1N1	Reduced lung injury and pro-inflammatory cytokine	(22)

## Data Availability Statement

All datasets presented in this study are included in the article/supplementary material.

## Author Contributions

All authors listed have made a substantial, direct and intellectual contribution to the work, and approved it for publication.

## Conflict of Interest

The authors declare that the research was conducted in the absence of any commercial or financial relationships that could be construed as a potential conflict of interest.
